# Recent Progress on Nanocrystalline Metallic Materials for Biomedical Applications

**DOI:** 10.3390/nano12122111

**Published:** 2022-06-19

**Authors:** Huafang Li, Pengyu Wang, Cuie Wen

**Affiliations:** 1 School of Materials Science and Engineering, University of Science and Technology Beijing, Beijing 100083, China; beskshark@163.com; 2 School of Engineering, RMIT University, Melbourne, VIC 3001, Australia; cuie.wen@rmit.edu.au

**Keywords:** biocompatibility, biomedical applications, corrosion behavior, mechanical properties, nanocrystalline metallic materials

## Abstract

Nanocrystalline (NC) metallic materials have better mechanical properties, corrosion behavior and biocompatibility compared with their coarse-grained (CG) counterparts. Recently, nanocrystalline metallic materials are receiving increasing attention for biomedical applications. In this review, we have summarized the mechanical properties, corrosion behavior, biocompatibility, and clinical applications of different types of NC metallic materials. Nanocrystalline materials, such as Ti and Ti alloys, shape memory alloys (SMAs), stainless steels (SS), and biodegradable Fe and Mg alloys prepared by high-pressure torsion, equiangular extrusion techniques, etc., have better mechanical properties, superior corrosion resistance and biocompatibility properties due to their special nanostructures. Moreover, future research directions of NC metallic materials are elaborated. This review can provide guidance and reference for future research on nanocrystalline metallic materials for biomedical applications.

## 1. Introduction

Biomedical metallic materials have good mechanical properties, biocompatibility, etc. However, the properties of conventional coarse-grained (CG) metallic materials do not fully meet clinical requirements. Nanocrystalline (NC) metallic materials have substantially improved mechanical properties, corrosion behavior, and biocompatibility than conventional CG metallic materials [1,2], thus it is obvious that the properties of nanocrystalline metallic materials can better meet clinical needs compared with conventional coarse-grained metallic materials. Therefore, NC metallic biomaterials are receiving increasing attention in recent years [3]. NC metallic biomaterials can be divided into two categories: bioinert NC metallic materials and biodegradable NC metallic materials. Currently, interest in bioinert NC metallic materials include NC pure titanium (Ti) and its alloys [4,5,6], NC SMAs [7], NC SS [8], and NC biodegradable metallic alloys including Fe-based alloys and Mg-based alloys [9]. The nanostructured metallic alloys open new avenues and concepts for medical implants, providing benefits in all areas of medical device technology.

Data from several studies suggest that after NC processing, NC metallic biomaterials have desirable characteristics, such as good manufacturability, and superior physical and mechanical properties [10,11]. This review summarizes the mechanical properties, corrosion behavior, biocompatibility, and clinical applications of bioinert NC metallic materials and biodegradable NC metallic materials in recent years and provides future directions for the development of biomedical NC metallic materials.

## 2. Bio-Inert NC Metallic Materials

### 2.1. Biomedical NC Pure Ti and Its Alloys

The excellent biocompatibility of medical devices manufactured from conventional CG pure Ti and its alloys for clinical applications has been demonstrated in several clinical investigations [12,13,14]. However, the mechanical strength and hardness of commercial CG Ti and its alloys need to be further improved. Nano-structuring of pure Ti and its alloys have recently been demonstrated to be a new horizon and promising alternative method for increasing the mechanical characteristics of conventional CG Ti alloys.

Researchers find that the mechanical performance of NC pure Ti is superior to that of conventional commercial CG Ti [15]. The strength of the NC Ti is nearly twice than that of conventional CG Ti: the σ_YS_ and σ_UTS_ of CG pure Ti were tested to be about 530 MPa and 700 MPa, respectively; after equal-channel angular pressing (ECAP), the CG Ti transformed to NC Ti and showed much higher σ_YS_ of 1267 MPa and σ_UTS_ of 1330 MPa [16]. In another study, the NC Ti-15Mo alloy showed significantly enhanced elastic modulus, microhardness, and tribological properties compared to its CG counterpart because of its higher relative density, sealed porosity, and grain size refinement. The binary NC Ti-15Mo alloy exhibited a microhardness of 315 HV_0.02_ and a modulus of elasticity of 95 GPa at 1373 K [17]. NC Ti-20Nb-13Zr has an average grain size of 70–140 nm with a duplex microstructure of the α-Ti (hcp) region surrounding the β-Ti (bcc) matrix, leading to a hardness of 660 HV, and the NC alloy also showed stimulation of new bone formation [18]. The σ_YS_ and σ_UTS_ of the NC Ti-5Ta-1.8Nb were reported to be 800 MPa and 750 MPa, respectively, and the fracture surface of the NC alloy exhibited shear bands and more ductile dimples compared to its CG counterpart which showed dimples and microvoids [19].

Not only have the strength and hardness of the NC Ti alloys increased, but their elongation has also been improved considerably compared to their CG counterparts. Shahmir et al. [20] obtained a good combination of strength and elongation in high-pressure torsion (HPT)-processed NC Ti at elevated temperatures ranging from 573 to 773 K. The strength, microhardness, and elongation of the NC pure Ti (Grade 2) with a grain size of 70 nm were measured as 945 MPa, 300 HV, and 9% at room temperature and approximately 350 MPa, 230 HV, and 130% at 673 K, respectively. Filho et al. [15] fabricated NC pure Ti (grade 2) with ECAP followed by cold rolling and found that this method gives the best strength-ductility combination. González-Masís et al. found that the nanocrystalline Ti-Nb-Zr-Ta-Hf had a combination of high hardness of 564 HV and moderate elastic of 79 GPa [21]. The mechanical properties of NC Ti better meet the requirements of bone replacement and repair. Two main factors can be identified to determine the strengthening mechanisms of NC metallic materials: grain refinement and increased dislocation density induced by the processing of the NC materials [22].

Elias et al. [23] compared the compressive and fatigue strength of dental implants made from the NC Ti (grade 4) processed by ECAP and the microcrystalline Ti (grade 4) and found that the NC Ti implants exhibited both higher compressive and fatigue strength for 5 × 10^6^ cycles than the microcrystalline Ti implants, and the ECAP-processed NC Ti exhibited transgranular fracture with no striation at the fatigue crack initiation and propagation regions. The increased compression and fatigue strengths of the NC Ti make it a very good material for dental implant applications. Javadhesari et al. produced Ti-50 at%Cu alloy which showed excellent mechanical properties: ultra-high microhardness of 10 GPa and acceptable toughness of 8.14 MPa⋅m^1/2^ [24]. All the abovementioned studies have demonstrated that NC Ti and its alloys have better mechanical properties for implant applications.

**Figure 1:**
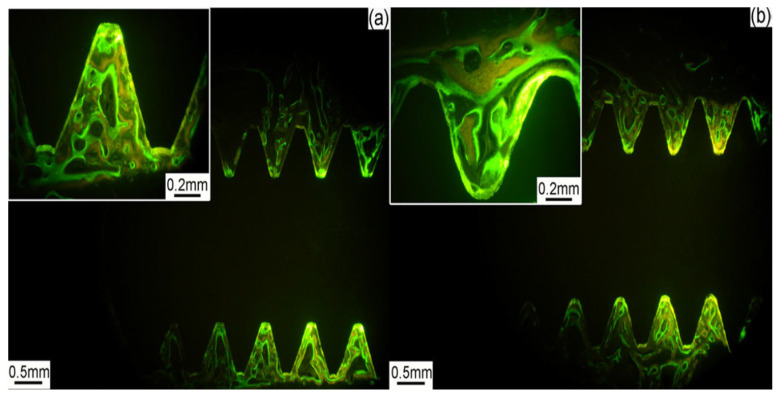
Histotomy of bone contact of: (**a**) CG Ti, and (**b**) NC Ti at four weeks, illustrated by fluorescence-dyeing reagents. Reprinted with permission from Ref. [25]. Copyright 2012, John Wiley and Sons.

In addition to the enhanced mechanical properties, an enhanced biological response can also be anticipated from NC metallic materials. For instance, the fibroblast mice cells L929 covered 53% commercially pure Ti (CP-Ti) surface and 87.2% nanostructured Ti surface after 72 h of cell culturing, indicating superior cytocompatibility of the NC Ti compared to its CG counterpart [4]. In another study, NC Ti showed an improvement in in vitro biosafety and long-term cellular functionalization in cytobiology and in vivo biostability [25]. Figure 1 shows the histotomy of bone contact of NC Ti at four weeks implantation as compared to CG Ti, indicating a higher osseointegration ability with freshly generated bone development direction and kinetics following implantation of NC Ti. Cell adhesion and proliferation test for nanocrystalline Ti25Nb16Hf showed lower adhesion and higher proliferation when compared to Ti grade 2 [26].

NC Ti-29Nb-13Ta-4.6Zr (the so-called TNTZ alloy) showed greater hardness than its CG counterpart, which is up to 310 HV [27]. Lin et al. [28] reported that the β Ti-35Nb-3Zr-2Ta alloy exhibited ultrafine equiaxed grains of approximately 300 nm after ECAP for 4 passes at 500 °C; the ECAP-processed alloy showed a longitudinal microhardness of 224 HV, a σ_YS_ of 390 MPa, and a σ_UTS_ of 765 MPa, while maintaining a good level of elongation of 16.5% and elastic modulus of 59 GPa. In another study, the microstructure of the HPT-processed TNTZ exhibited a single phase of β grains with diameters of a few hundred nanometers and high-angle boundaries, and due to the severe plastic deformation of the HPT process, the grains exhibited non-uniform subgrains with high dislocation density. The tensile strength of the nanocrystalline TNTZ alloy increased significantly [29]. In summary, the microhardness of NC TNTZ alloy is consistently much higher than its CG counterpart [30].

Xie et al. [31] reported that HPT-processed NC β-Ti alloy (Ti-36Nb-2.2Ta-3.7Zr-0.3O, at.%) showed markedly improved mechanical and biocompatibility properties; the hardness and elastic modulus of the NC Ti alloy were, respectively, 23% higher and 34% lower than those of its CG counterpart. The decrease in the elastic modulus of a metallic implant biomaterial is of critical significance because it helps prevent stress shielding that occurs when the implant is stiffer than its surrounding host bone. The β-type Ti-24Nb-4Zr-8Sn alloy processed by warm swaging and warm rolling with a uniform microstructure comprising a β phase with a size less than 200 nm and the precipitation of nanosized α phase, exhibited high ultimate tensile strength of 1150 MPa, low elastic modulus of 56 GPa, and good ductility with an elongation of 8%, along with large-scale nonlinear deformation behavior with a recoverable strain of up to 3.4% [32]. Kent et al. [33] reported the mechanical properties of the Ti-25Nb-3Zr-3Mo-2Sn alloy processed by a modified accumulative roll bonding (ARB) technique, and found that after 4 cycles of rolling, the ARB-processed sample exhibited significantly refined β grains heavily elongated in the rolling direction and NC α precipitates distributed on the β grain boundaries, with an ultimate tensile strength of 1220 MPa, a 0.5% proof stress of 946 MPa, which were, respectively, ~70% higher and almost double those of the CG solution treated counterpart. He et al. [34] investigated the mechanical properties of the Ti_60_Cu_14_Ni_12_Sn_4_M_10_ (M = Nb, Ta, Mo) alloys prepared using arc melting and copper mold casting. The alloys exhibited a composite microstructure containing a micrometer-sized dendritic β-Ti(M) phase dispersed in an NC matrix with a compressive elastic modulus in the range of 59–103 GPa, a compressive yield strength in the range of 1037–1755 MPa, and a compressive plastic strain in the range of 1.68–21.34% [35].

Some studies have also shown that NC Ti alloys have superior electrochemical corrosion resistance. Yilmazer et al. [36] evaluated the corrosion behavior of the HPT-processed Ti-29Nb-13Ta-4.6Zr alloy in simulated body fluid (SBF) using electrochemical impedance spectroscopy (EIS) and found that the NC alloy exhibited improved corrosion performance than its CG counterpart due to a passivated surface layer contained a titania (TiO_2_) matrix dispersed with zirconia (ZrO_2_), niobia (Nb_2_O_5_), and tantala (Ta_2_O_5_) oxides. Furthermore, an NC Ti alloy exhibits better hydrophilic property due to the NC structure [37]. This will lead to improved protein adsorption properties because of the increased contact points between the protein and the NC surface. The adsorption of bone morphogenetic proteins on a material surface affects the cell adhesion, spreading, and proliferation on the material. M. A. Hussein showed that nanocrystalline Ti20Nb20Zr alloy had a hydrophilic nature compared with a CP Ti [38]. Xie et al. [31] showed that HPT-processed NC β-type Ti-36Nb-2.2Ta-3.7Zr-0.3O (at.%) showed enhanced cell attachment and proliferation of human gingival fibroblasts (HGF) after 30 min cell seeding compared to its CG counterpart.

### 2.2. Biomedical NC SMAs

Nitinol refers to a family of SMAs composed of nickel (Ni) and Ti with a unique combination of properties including superelasticity and shape memory properties. The word nitinol is originated from its composition (Ni-Ti) and its place of discovery of Naval Ordnance Laboratory (USA) by William J. Buehler and Frederick Wang in 1963 [39]. Nitinol are the most widely used biomedical SMAs due to their large recoverable strains (~8%) in polycrystalline forms [40]. The phase transformation temperature of medical Ni-Ti SMAs is close to that of the human body; therefore, this class of Ti alloys plays an important role in the medical field that is unmatched by other materials. Conventionally, Ni-Ti SMAs are frequently used in orthopedics, dentistry, and cardiovascular stent treatments. NC Ni-Ti-based SMAs offer even better overall mechanical properties [41]. Ti-Zr-based alloys alloyed with Nb, Ta, Mo, and Sn are also another type of shape memory alloys. Sheremetyev et al. [42] reported that Ti-18Zr-15Nb alloy processed by ECAP at 250 °C for 7 passes showed a σ_YS_ of 962 MPa, a σ_UTS_ ultimate tensile strength of 988 MPa, and an elongation of 5.4%.

Yan et al. [43] found that compared to CG NiTi SMAs, the NC austenite NiTi (Ni-49.3Ti, at.%) SMA with a B2 (CsCl) type ordered structure showed a significantly enhanced compressive yield strength of 2552.1 MPa, and the value of stress-induced austenite transformation increased with decreasing grain size. Nie et al. [44] found that the HPT-processed NC Ni_50.2_Ti_49.8_ alloy exhibited a hardness of 456.8 ± 14.9 HV. Sharifi and Kermanpur [45] performed hot rolling and annealing at 900 °C on Ni_50_Ti_50_ alloy, followed by cold rolling with thickness reduction of 70% and annealing at 400 °C, and the resultant NC alloy exhibited superelastic properties including a recoverable strain of 12% and an upper plateau stress (σ_SIM_) (i.e., the critical stress for stress-induced martensitic transformation) of 610 MPa, which is significantly higher than that of the CG alloy with a σ_SIM_ of 160 MPa. Baigonakova et al. [46] found that NiTi0.1Ag wires provided optimal strength (1450 MPa) and ductility (33.4%) properties, due to the dislocation-free homogeneous nanocrystalline structure.

**Figure 2:**
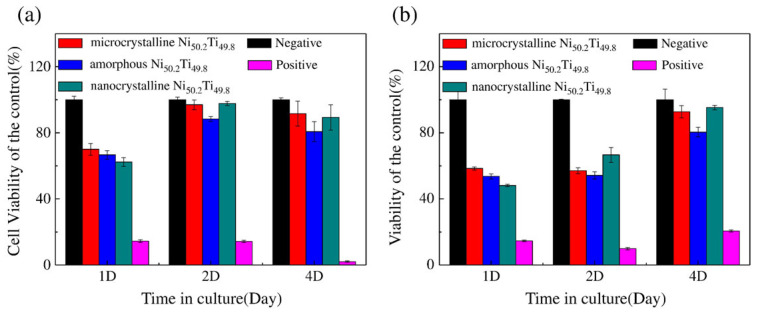
Cytotoxicity of (**a**) L-929 and (**b**) MG63 cell lines co-cultured with extracts from microcrystalline Ni_50.2_Ti_49.8_, and nanocrystalline Ni_50.2_Ti_49.8_. Reprinted with permission from Ref. [44]. Copyright 2010, Elsevier.

However, NiTi SMAs contain a large amount of Ni ions. NiTi alloy implants might release Ni ions into the human tissue and cause severe allergic reactions after implantation in the body [47]. Nanocrystallization not only significantly improved the mechanical and superelastic properties of NiTi SMAs, but also fundamentally solved the problem of rapid Ni ion release because NC NiTi SMAs improved corrosion resistance compared to their CG counterparts. Shri et al. [48] found that through severe plastic deformation of HPT, the corrosion behavior of NiTi SMAs was changed by grain refinement, the NC Ti-50Ni (at.%) exhibited a stable, protective layer on its surface in a cell culture medium and increased corrosion resistance, leading to decreased Ni ion release. Nie et al. [44] reported superiorly higher corrosion resistance of HPT-processed NC Ni_50.2_Ti_49.8_ alloy with a substantially lower rate of Ni ion release than its microcrystalline counterpart in both Hanks’ solution and artificial saliva, which was far below the threatening threshold of a daily diet. The results of murine fibroblast (L-929) and osteoblast cell lines (MG63) cultured and cell proliferation are shown in Figure 2. There is no cytotoxicity for nanocrystalline Ni_50.2_Ti_49.8_ till 4 days culture with L-929 and MG63.

Li et al. [7] investigated the in vitro and in vivo biological properties of an ECAP-processed NC Ti_49.2_Ni_50.8_ alloy for orthopedic implant applications and indicated enhanced cell viability, adhesion, proliferation, ALP (alkaline phosphatase) activity, and mineralization than its CG counterpart. 

### 2.3. Biomedical NC Stainless Steels (SSs)

Biomedical NC SSs have enhanced passivation behavior and corrosion resistance than their CG counterparts; furthermore, NC SSs also exhibit enhanced hydrophilic and protein adsorption properties, leading to improved biological properties including better cell attachment, spreading, and proliferation [49].

**Figure 3:**
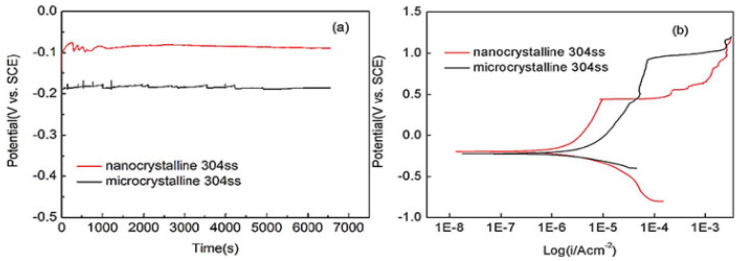
The electrochemical curves of (**a**) OCP and (**b**) polarization of microcrystalline 304 SS and nanocrystalline 304 SS in artificial saliva. Reprinted with permission from Ref. [49]. Copyright 2011, Elsevier.

Previous studies have found that NC ASTM F-138 austenitic SSs have higher mechanical strength than their CG counterpart [15,50,51]. Heidari et al. found that the ASTM F2581 nanocrystalline stainless steels had 824 MPa yield strength, and the strength exceeded 1 GPa [51]. NC 304 SS fabricated by severe rolling showed a hardness of 480.0 ± 10.1 HV and improved corrosion resistance; Figure 3a shows the OCP curves of nanocrystalline 304 SS and microcrystalline 304 SS in artificial saliva. The result of OCP curve indicates that nanocrystalline stainless steels show more corrosion resistance than microcrystalline stainless steels in artificial saliva. Polarization studies (Figure 3b) revealed that NC 304 SS are more corrosion resistant than conventional CG 304 SS in an oral-like environment with higher corrosion potential [49].

Nie et al. using electrochemical measurement concluded a similar result that nanocrystalline stainless steel has higher corrosion resistance compared to microcrystalline stainless steel and the corrosion behavior of nanocrystalline 304 SS do not have significantly superior resistance to pitting corrosion compared to microcrystalline stainless steels [52].

### 2.4. Other Types of Bio-Inert NC Metallic Materials

**Figure 4:**
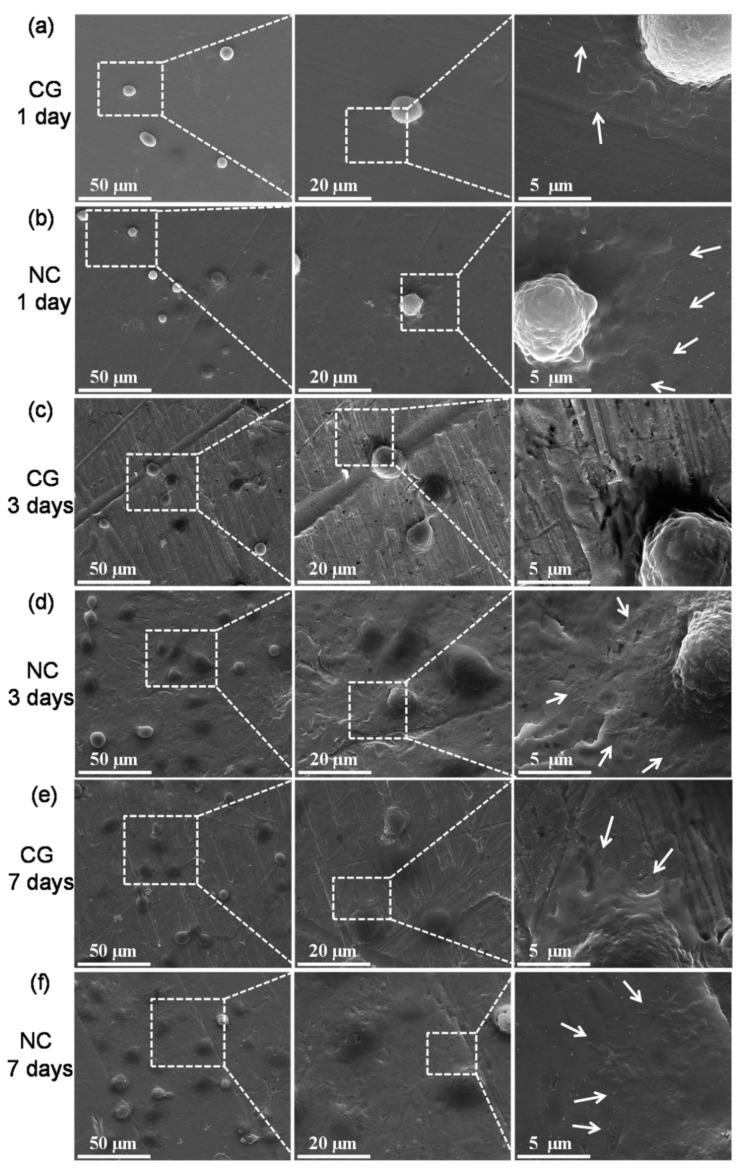
Typical morphologies of hFOBl.19 cells cultured on: (**a**,**c**,**e**) CG and (**b**,**d**,**f**) NC Ta surfaces for (**a**,**b**) 1 day, (**c**,**d**) 3 days, and (**e**,**f**) 7 days. Arrows indicate filopodia extensions. Reprinted with permission from Ref. [55].

There are also some other types of NC metallic materials for biomedical application. NC silver (Ag) prepared by spark plasma sintering (SPS) at 600 K for 5 min exhibited a mean grain size of 380 nm and showed a σ_YS_ 4.6 times higher than that of CG counterpart with a mean grain size of 49.65 µm and more than 30% uniform elongation [53]. The HPT-processed nanocrystalline CoCrMo exhibited improved tribocorrosion resistance but deteriorated corrosion resistance [54]. Huo et al. [55] fabricated an NC surface with an average grain size of ≤20 nm using sliding friction treatment (SFT) on CG pure Ta and comparatively studied the osteoblast cell responses to the CG and NC Ta using human osteoblastic hFOBl.19 cells. Their results showed that the NC surface exhibited higher surface hydrophilicity and enhanced corrosion resistance than the CG surface, thus leading to enhanced osteoblast adherence and spreading after 1 day’s cell culturing and markedly improved cell proliferation, maturation, and mineralization after 14 days’ cell culturing. Figure 4 shows the morphologies of hFOBl.19 cells on CG and NC pure Ta after 1, 3, and 7 days’ of culturing. The results of this study indicated the superior cytocompatibility of the NC Ta surface compared to its CG counterpart [55].

Some bio-inert NC metallic biomaterials published in the literature in recent years, their fabrication methods, grain sizes, mechanical properties, corrosion behaviors, biocompatibility, and potential applications are summarized in Table 1.

## 3. Biodegradable NC Metallic Materials for Biomedical Applications

Biodegradable metals have been research hotspots for the last two decades [62]. Nanocrystallization of biodegradable metals can further enhance their comprehensive properties. Wang et al. [63] performed hot rolling on Mg–2Zn alloy and found that the alloy exhibited a grain size of ~70 nm with a high σ_YS_ of 223 MPa and σ_UTS_ of 260 MPa, and a strong corrosion resistance with a corrosion rate of 0.2 mm/y in vivo when tested using Sprague Dawley rats. Nie et al. [9] demonstrated that ECAP-processed NC pure iron (Fe) exhibited higher corrosion resistance and improved hemocompatibility and cytocompatibility. Zhang et al. [64] investigated the corrosion resistance of an NC Mg-2Zn-0.24Ca (wt.%) alloy processed by HPT and annealing and reported that the grain size, number of (0002) oriented grains, second phase, and surface stress of the alloy changed with the annealing temperature and time, and these factors affected the corrosion rate. The HPT-processed alloy showed the best corrosion resistance with the maximum polarization resistance and lowest hydrogen evolution rate when annealed at 210 °C for 30 min.

**Figure 5:**
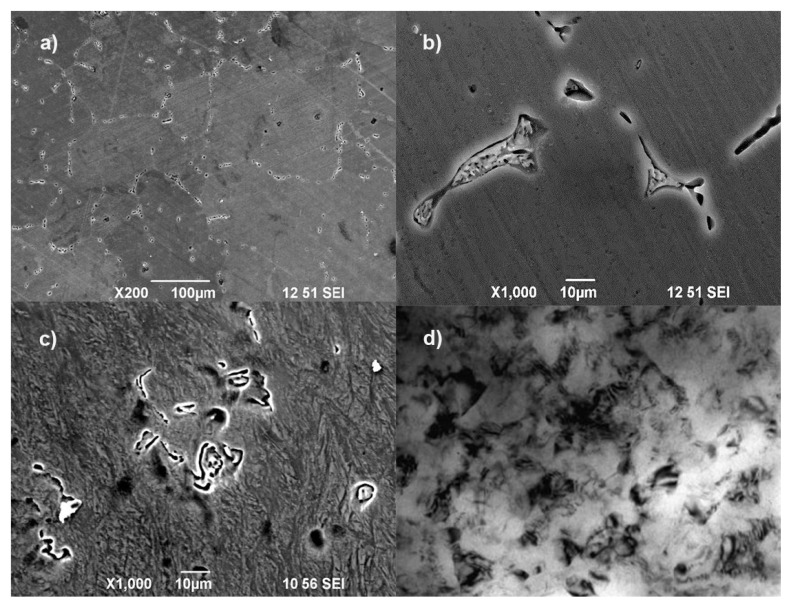
Microstructures of Mg-1Ca alloy: (**a**,**b**) scanning electron microscopy images of CG alloy; (**c**,**d**) transmission electron microscopy images of NC alloy. Reprinted with permission from Ref. [65].Copyright 2020, Elsevier.

The HPT-processed NC Mg-1Ca alloy showed at least 5-fold improvement in corrosion resistance than the CG alloy due to the separation of the second phase (Mg_2_Ca) particles and their continuous nanoprecipitation. Figure 5 shows the micrographs of CG and NC Mg-1Ca alloy. The microstructure of the homogenized CG Mg-1Ca alloy contained equiaxial grains (Figure 5a), while the NC alloy showed a much finer grain size of 100 ± 9 nm with a greater dislocation density and higher shear strength (Figure 5c) [65].

Gu et al. fabricated Mg-3Ca alloys with different grain sizes by melt-spinning at different wheel rotating speeds and their electrochemical test results showed that the corrosion rate of the NC Mg-3Ca in simulated body fluid was significantly reduced compared with CG Mg-3Ca, and the NC Mg-3Ca alloy showed a more uniform corrosion morphology. Further, the extract of the NC Mg-3Ca alloy showed no cytotoxicity in relation to L-929 cells, whereas the extract of CG Mg-3Ca alloy did. On the surface of the NC Mg-3Ca alloy, the L-929 cells exhibited improved adhesion than the CG Mg-3Ca alloy [66].

Table 2 summarizes some biodegradable NC metallic biomaterials published in the literature in recent years, their fabrication methods, grain sizes, and mechanical, corrosion, and biocompatibility properties. Most of the degradable nanocrystalline alloys reported thus far are Mg-based, with a small amount of Fe-based alloys; Zn-based alloys have not yet been publicly reported, and further development of Zn-based nanocrystalline alloys is needed in the future.

## 4. Conclusions

This article provides a review on the mechanical properties, corrosion behavior, biocompatibility, and clinical applications of different NC metallic biomaterials. The main conclusions are as follows:

1. Biomedical NC metallic materials, such as Ti and its alloys, TNTZ, NiTi SMAs, SS, and biodegradable Fe and Mg alloys have significantly improved tensile yield strength, ultimate tensile strength, and hardness without significant reduction in ductility.
2. Biomedical NC metallic materials, such as Ti and its alloys, TNTZ, NiTi SMAs, SS, and biodegradable Fe and Mg alloys have better corrosion resistance than their conventional CG metallic materials.
3. Nanocrystallization of metallic biomaterials can improve their biocompatibility due to the unique nanostructures on their surfaces.
4. In future research, the relationships between grain size, microstructural characteristics, and material properties of NC metallic materials should be systematically investigated. More research and development should be devoted to zinc-based degradable NC alloys, iron-based degradable NC alloys, and Mg and plural NC alloys.

## Figures and Tables

**Table 1:** Summary of bio-inert nanocrystalline metallic biomaterials.

Materials	Methods	Grain Size (nm)	Mechanical Properties	Corrosion Properties	Biocompatibility	Application	Ref.
Pure Ti (grade 2)	ECAP + cold rolling	About 500	σ_YS_: Increased to 796 MPa σ_UTS_: Increased to 876 MPa Hardness: Increased to 271 HV	-	-	Bone replacement and repair	[15]
Pure Ti (grade 4)	ECAP	150	Good plasticity under compression Fatigue strength higher than conventional CG Ti G4	-	-	Dental implant	[23]
Pure Ti (grade 4)	SPD+TMT	150	σ_UTS_: 1240 MPa σ_YS_: 1200 MPa Elongation: 12% Fatigue strength at 10^6^ cycles: 620 MPa	-	(Fibroblast mice cells L929) Occupied surface after 72 h conventional CP Ti: 53.0% NC CP grade 4: 87.2%	Dental implant	[4]
CP Ti (grade 4)	ECAP	250	Ductility: 5% TS: 1190 MPa σ_UTS_: 1240 MPa Elongation: 11.5% Fatigue strength at 10^6^ cycles: 620 MPa	-	Protein adsorption: Better than CP Ti L929: Cell viability increases and growth ahead MG63: Grow well VSMCs: Not proliferate well ECV304: Grow well Hemolysis rates: Less than 5% In vivo test: Superior bone formation	Bone replacement	[25]
Pure Ti (grade 2)	HPT	70	Strength: 940 MPa High hardness: 300 HV Elongation: 130%	-	-	-	[20]
Pure Ti (grade 2)	ECAE	300	σ_YS_: 620 MPa Ductility: 21%	-	-	-	[22]
Pure Ti (grade 4)	ECAE	300	σ_YS_: 758 MPa Ductility: 25%	-	-	-	[22]
Pure Ti	ECAP-conform + Drawing	-	σ_UTS_: 1330 MPa σ_YS_: 1267 MPa Elongation: 11% Endurance limit: 10^7^ cycles of 620 MPa	-	-	Dental implants	[16]
B2 austenite NiTi shape memory alloy (Ni-49.3 at.%Ti)	SPD. + Annealing 4 h	45	Compressive yield stress: 2552.1 MPa Fracture strain decreased 11.7% σ_SIM_: 267.8 MPa	-	-	-	[43]
Ti-15Mo	High energy ball mill + Hot isostaticallypressed	29 (1373 K)	Microhardness: 315 HV_0.02_ (1373 K) Elastic modulus: 95 GPa Friction coefficient: 023–0.35 (1373 K)	-	-	-	[17]
Ti-50at.%Ni	HPT	-	-	Increase corrosion resistance in the cell culture medium (stable and protective passive film)	-	-	[48]
Ni_50_Ti_50_	70% cold rolling + annealing at 400 °C for 1 h	20–70	σ_SIM_: 610 MPa	-	-	-	[45]
Ni_50.2_Ti_49.8_	HPT	-	Hardness: 456.8 ± 14.9 HV	Superiorly higher corrosion resistance than microcrystalline Ni50.2Ti49.8 (Hanks’ solution and artificial saliva)	L-929: No cytotoxicity MG63: No cytotoxicity	-	[44]
Ti_49.2_Ni_50.8_	ECAP	150–250	-	-	Hemolysis rates: 0.1% Number of adhered platelets: Lower than microcrystalline Cell viability: Higher Better osteogenesis functions In vivo: Enhanced cell viability, adhesion, proliferation, ALP activity, and mineralization, and increased periphery thickness of new bone	Orthopedic biomaterials	[7]
Ti-6Al-4Fe	Mechanical alloying	-	Hardness: 335 ± 17 HV_0.05_ (powders milled for 2 h), 387 ± 19 HV_0.05_ (powders milled for 6 h), 475 ± 23HV_0.05_ (powders milled for 12 h), 660 ± 33 HV_0.05_ (powders milled for 18 h) Young’s modulus: 110–197 GPa	-	-	Bone replacement	[56]
Ti-5Ta-1.8Nb	Cryo-rolling	20	σ_YS_: About 800 MPa σ_UTS_: ~750 MPa Elongation: About 5%	-	-	-	[19]
Ti13Nb13Zr	SPD	200	Young’s modulus: 72 GPa σ_YS_: 1150 MPa Hardness: 300 HV	-	-	Dental implant	[57]
Ti-18Zr-15Nb	ECAP at 250 °C for 7 passes	20–100	σ_YS_: 962 MPa σ_UTS_: 988 MPa Ductility: 5.4%	-	-	-	[42]
Ti-20Nb-13Zr	SPS	-	Hardness: 660 HV	-	Stimulate new bone formation	Dental and orthopedic applications	[18]
Ti-13Ta-xSn (x = 3, 6, 9 and 12, at.%)	Mechanical alloying	10	-	-	-	-	[58]
Ti25Nd16Hf	Cold rolling at 95% reduction	50	Ductility: 4.0% σ_YS_: 790 MPa σ_UTS_: 870 MPa Elastic modulus: 42.3 GPa	The highest corrosion resistance (corrosion current density 1.52 μA/cm^2^) compared with Ti25Nb16Hf (0% C.R.) and Ti grade II	Cytotoxicity: ExcellentCell adhesion (MG63 cells): Lower than pure Ti Cell proliferation: Properly	-	[26]
TiNbZrTaHf	HPT	<100	Hardness: 564 ± 22 HV Elastic modulus: 79 ± 3 GPa Good plasticity under localized compression	-	-	-	[21]
Ti-29Nb-13Ta-4.6Zr	HPT	40–500	Ductility: Decrease σ_UTS_: Increase Hardness: Great	-	-	-	[29]
Ti-35Nb-3Zr-2Ta	ECAP	300–600	Ductility: 16% σ_UTS_: 765 MPa Elastic modulus: 59GPa	-	-	-	[28]
Ti-24Nb-4Zr-8Sn	Warm swaging and warm rolling	-	Recoverable strain: 3.4%σ_UTS_: 1150 MPaElastic modulus:56 GPaDuctility: 8%	-	-	-	[32]
Ti-29Nb-13Ta-4.6Zr	HPT	-	σ_UTS_: 800–1100 MPa Elongation: 7% Young’s modulus: 60 MPa	-	-	-	[59]
Ti-29Nb-13Ta-4.6Zr (TNTZ)	HPT	-	Hardness: Higher than 310 HV	-	-	-	[27]
Ti-29Nb-13Ta-4.6Zr	HPT	-	Hardness: >183 HV (Hardness values of peripheral region higher than that of central region)	-	-	-	[30]
Ti-36Nb-2.2Ta-3.7Zr-0.3O	HPT	-	Elastic modulus: 43 GPa (30% lower than CG counterpart) Hardness: 320HV (23% higher than CG counterpart)	-	Human gingival fibroblasts: Attachment and proliferation were enhanced Human dental follicular Cells: Higher cell density	-	[31]
Ti-25Nb-3Zr-3Mo-2Sn	Accumulative roll bonding	130	σ_UTS_: 1220 MPa 0.5% proof stress: 946 MPa Ductility: 4.5%	-	-	-	[33]
Ti_60_Cu_14_Ni_12_Sn_4_Nb_10_	Arc melting and copper mold casting	-	σ_YS_: 1052 MPa Young’s modulus: 59 GPa Strain at yield point: 2.1%	-	-	-	[35]
Ti _60_Cu_14_Ni_12_Sn_4_M_10_ (M = Nb, Ta, Mo)	Arc melting and copper mold casting	-	σ_YS_: 1037–1755 MPa Young’s modulus: 59–103 GPa Plastic strain: Up to 21%	-	-	-	[34]
Ti/1.3HMDS	Powder metallurgy	365	Hardness: 320 HVYoung’s modulus: 129 MPa σ_YS_: 1439 MPa Breaking elongation: 7.1%	Osteogenically induced hMSC: Comparable with CP Ti and Ti6Al4V	-	Bone repair	[60]
ASTM F-138 austenitic steel	ECAP + cold rolling	100–200	YS: Increased to 1055 MPa σ_UTS_: Increased to 1059 MPa Hardness: Increased to 339 HV	-	-	Bone replacement and repair	[15]
Ti-Cu	Mechanical alloying and sintering		Hardness: 10 GPa	The corrosion behavior of the alloy was slightly lower than cp-Ti	98% anti-bacterial rate against Staphylococcus aureus (*S. aureus)* and Escherichia coli (*E. coli)*, excellent cell viability to MG-63 cells, and high osteoblast formation rate	Orthopedic material	[24]
304 stainless steel	Severe rolling	-	Strength 1280 MPa (NC 304), 640 MPa (CG 304 SS)	More corrosion resistant than the microcrystalline 304 SS in artificial saliva	Cytotoxicity (murine fibroblast cells): Better than microcrystalline 304 SS	-	[49]
304 stainless steel	Severe rolling	50	Hardness: 480.0 ± 10.1 HV	Better corrosion resistance (Hanks’ solution)	Cytotoxicity (L-929, NIH 3T3): No toxic effect, Low hemolysis rate	-	[52]
316L stainless steel	Severeplastic deformation	Around 5 at the surface	Maximum nanohardness: 6.2 GPa Young’s modulus: 200–220 GPa	-	-	-	[50]
Stainless steel	ECAP	74 (strain-induced martensite, BCC); 31 (austenite, FCC)	-	-	-	-	[61]
Austenitic stainless steel	Binder assisted extrusion	-	Compressive yield strength: 824 MPa Compressive strength: 1326 MPa Uniform elongation: 50% Hardness: 339 HV	-	-	-	[51]
Pure silver	Spark plasma sintering (sintered 600 K for 5 min)	300	σ_YS_: 146 MPa Uniform elongation: 30%	-	-	-	[53]
CoCrMo	Five-turns HPT	-	Compressive yield strength: 1.25 GPa Hardness: 9.3 GPa Elasticity modulus: 203 GPa	Reduce corrosion resistance	Improved tribocorrosion resistance	Hip and knee replacements	[54]

**Table 2:** Summary of biodegradable nanocrystalline metallic materials.

Materials	Methods	Grain Size (nm)	Mechanical Properties	Corrosion Properties	Biocompatibility	Ref.
Pure Fe	ECAP (8 passes)	-	σ_UTS_: 470 MPa (double of CG counterpart) Hardness: 444 ± 31 kg f mm^−2^ (4 times of CG counterpart)	Higher corrosion resistance	Better hemocompatibility: Hemolysis less than 5% VSMCs: Inhibited (less than 60%) ECs and L929: Improved (more than 80%)	[9]
Mg-2wt.%Zn	Hot-rolled	70	σ_YS_: 223 MPa σ_UTS_: 260 MPa	Good corrosion resistance (corrosion rate in vivo: 0.2 mm/y)	-	[63]
Mg-1Zn-1Mn-0.3Zr	20 h Ball milling + annealing	45	-	Corrosion resistance in Ringer solution improved	-	[67]
Mg-Zn-Ca	HPT	With an increase in annealing temperature, grain size increased from 100 to 900		Corrosion resistance increases with annealing temperature increased from 90–210 °C Corrosion resistance decreases with temperature increased after 210 °C	-	[64]
Mg-1Ca	HPT + Annealing	100	-	Increased corrosion resistance	-	[65]
Mg-3Ca	Melt-spinning	200–500	-	Uniform corrosion morphology	No toxicity and improved adhesion in relation to L-929 cells	[66]
